# Coinfections and differential diagnosis in immunocompetent patients with uveitis of infectious origin

**DOI:** 10.1186/s12879-018-3613-8

**Published:** 2019-01-25

**Authors:** Alejandra de-la-Torre, Juanita Valdés-Camacho, Clara López de Mesa, Andrés Uauy-Nazal, Juan David Zuluaga, Lina María Ramírez-Páez, Felipe Durán, Elizabeth Torres-Morales, Jessica Triviño, Mateo Murillo, Alba Cristina Peñaranda, Juan Carlos Sepúlveda-Arias, Jorge Enrique Gómez-Marín

**Affiliations:** 1 Unidad de Inmunología, Grupo de Investigación en Neurociencias (NeURos), Escuela de Medicina y Ciencias de la Salud, Universidad del Rosario, Carrera 24 # 63 C 69, Bogotá, Colombia; 2 Departamento de Investigación, Escuela Superior de Oftalmología-Instituto Barraquer de América, Bogotá, Colombia; 3 GEPAMOL, Centro de Investigaciones Biomédicas, Facultad de Ciencias de la Salud, Universidad del Quindío, Armenia, Colombia; 4 Grupo de Investigación Infección e Inmunidad, Facultad de Ciencias de la Salud, Universidad Tecnológica de Pereira, Pereira, Colombia

**Keywords:** Diagnosis, Co-infections, Immunocompetent, Goldmann-Witmer, PCR, Ocular toxoplasmosis

## Abstract

**Background:**

Making a definite diagnosis of infectious uveitis is a challenging task because many other infectious, and non-infectious uveitis, may have similar non-specific symptoms and overlapping clinical appearances. Co-infections in immunocompetent patients are not frequently proved with traditional serologic-diagnostic tools.

**Methods:**

Descriptive transversal study, in a Uveitis Service of an Ophthalmology Reference Center, in Bogotá, Colombia, from July 2014 to February 2016. Aqueous humor (AH) and/or vitreous fluid, blood and serum samples were collected from consecutive patients suspected of having infectious uveitis. The diagnosis of ocular toxoplasmosis (OT) was confirmed by the Goldmann–Witmer coefficient (GWC) and by polymerase chain reaction (PCR). Differential diagnosis by PCR in AH was done for viral origin such as Cytomegalovirus (CMV), Herpes simplex virus type 1 (HSV1), Herpes simplex virus type 2 (HSV2), Varicella zoster virus (VZV), Epstein-Barr virus (EBV) and *Mycobacterium tuberculosis*.

**Results:**

In 66 Colombian patients with uveitis of presumed infectious origin: 22 (33.3%) were confirmed as OT, 16 (24.2%) as undetermined OT, five (7.5%) as co-infections and 23 (34.8%) as other uveitis. *Toxoplasma* coinfection with *M. tuberculosis* was identified in one case by PCR and in four cases with HSV by GWC. The initial clinical diagnosis changed, after laboratory examination, in 21 cases (31.8%).

**Conclusions:**

Clinical diagnosis can be changed by laboratory examination in a significant proportion of cases of uveitis. Diagnosis of OT should combine the use of PCR and GWC to reach the maximum of confirmation of cases. The use of multiple laboratory methods is necessary to identify co-infections and viral infections that can mimic OT in immunocompetent patients.

**Electronic supplementary material:**

The online version of this article (10.1186/s12879-018-3613-8) contains supplementary material, which is available to authorized users.

## Background

*Toxoplasma gondii* is one of the most common human zoonosis, affecting about a third of the world’s population [1]. Around 10% of people that acquire this infection postnatally [2, 3], and up to 80% of children congenitally infected [4, 5], develop ocular toxoplasmosis (OT). This clinical form of toxoplasmosis is the most common etiology of posterior uveitis worldwide [1, 6].

Although in clinical practice a majority of cases of OT are diagnosed by a combination of consistent clinical features and supportive serological results [7], in cases of atypical presentations it is of utmost importance to differentiate OT from other causes of posterior uveitis that share similar clinical characteristics [8–14].

A definitive diagnosis is only obtained after direct evidence of the presence of the parasite in aqueous humor (AH) by polymerase chain reaction (PCR) that amplifies specific *Toxoplasma* DNA or by determining the eye’s own antibody production through Goldmann-Witmer coefficient (GWC) [15, 16]. These methods cannot only confirm the OT diagnosis but can also rule out other similar infectious diseases [17].

It has been described that the analysis of AH by PCR changed the diagnosis and treatment in more than a third of patients, and it should be considered for uveitis of an atypical clinical form, recurrent severe uveitis of unclear etiology, and therapy refractory cases [18]. As the relative importance of different etiologies changes from one geographical site to another, we aim to evaluate the differential diagnosis of this parasitic infection in immunocompetent patients seen in an Ocular Immunology and Uveitis Service, in Bogotá, Colombia. No previous description of this diagnostic approach has been presented in Latin America.

## Methods

### Purpose

To estimate the number of co-infections and infections by *Toxoplasma gondii*, *Mycobacterium tuberculosis* and Herpesvirus in Colombian immunocompetent patients with uveitis of presumed infectious origin.

### Population sample

A descriptive transversal study was carried out involving 66 patients of a Uveitis Service of an Ophthalmology Reference Center, *Clínica Barraquer*, in Bogotá, Colombia, from July 2014 to April 2016.

### Inclusion criteria and clinical data collection

The inclusion criteria were patients of all ages, presenting with uveitis of presumed infectious origin. A complete clinical history was taken and an ophthalmological examination was performed for all patients. Data collected included demographic features, age and sex, ophthalmic findings, which comprised affected eye, best corrected visual acuity (BCVA) using the Snellen Charts, intraocular pressure, slit-lamp examination findings, the grade of inflammation in the anterior chamber and in the vitreous according to the Standardization of Uveitis Nomenclature (SUN) guidelines, fundus examination findings with a description of retinochoroidal lesions, size, number and localization if present, and evaluation of ocular complications.

### Ethics approval and consent to participate

This study was conducted according to the tenets of the Declaration of Helsinki, strictly following the Guide for Good Laboratory Procedures. The protocol was approved by the Institutional Ethical Committees (Reference numbers: 5–14-1 from Universidad Tecnológica de Pereira and 030314 from Escuela Superior de Oftalmología - Instituto Barraquer de América) and all participants provided written informed consent.

### Laboratory analysis

AH and/or vitreous fluid, blood and serum samples were collected from consecutive patients suspected of having uveitis of infectious origin at presentation. The diagnosis of OT was confirmed by serum titers, quantification of antibodies with the GWC and by detection of *Toxoplasma gondii* genomes with PCR. Differential diagnosis by PCR in AH was done for viral origin and *Mycobacterium tuberculosis*. AH samples (0.1 to 0.2 ml) were obtained at the Ophthalmologic Center, *Clínica Barraquer*, in a surgery room, under sterile conditions after topical anesthesia, and were sent to a laboratory for analyses. For real-time PCR (qPCR) assays, DNA extraction was performed using the QIAamp DNA Mini Kit (Qiagen, Hilden, Germany) and procedures were performed as recommended by the manufacturer. DNA from a pellet of AH was obtained by incubating samples for 10 min at 56 °C with the cell lysis solution. After centrifuging at 6000 g for 1 min at 37 °C, flow through was discarded and the spin column was recovered. A two wash step was performed with washing solution and the cellular proteins were then eluted from the spin column. To detect *Toxoplasma* DNA in AH (0.1 to 0.2 ml), a qPCR TaqMan-based assay was used for this study, as described previously [19]. Briefly, this test amplifies a 100-bp of a 529-bp repetitive fragment (RE) that is reported to be repeated 300 times in the genome of *T. gondii* (Genebank accession number AF146527). The TaqMan probe TACAGACGCGATGCCGCTCC, and RE primers F- GCCACAGAAGGGACAGAAGT and R- ACCCTCGCCTTCATCTACAG, were redesigned using web-based software (found at https://www.genscript.com/ssl-bin/app/primer). The Taqman probe was labeled at the 5’with 6-carboxyfluorescein (FAM) and at the 3′ with non-fluorescent quencher. qPCR was performed using a Platinum® Quantitative PCR SuperMix-UDG (Invitrogen, Carlsbad, California, United States). The amplification protocol consisted of two initial stages of 50 °C for 2 min, held for UDG incubation, and 95 °C for 2 min, held for UDG inactivation, followed by 40 cycles of 95 °C for 15 s of denaturation, followed by 60 °C for 30 s of annealing and extension. The positive control was DNA from the RH strain and the negative control was distilled water in the presence of primers. Control for contamination during DNA extraction was also included and consisted of a tube without a template but containing all reagents for DNA extraction and filled with the same pipette. An additional control was a blood sample from a patient that tested negative for Immunoglobulin G (IgG) and Immunoglobulin M (IgM) *Toxoplasma* antibodies.

The presence of human herpes virus 3 (*VZV*) IE62 gene, *HVS2* UL36 region, human herpes virus 5 (*CMV*) glycoprotein B gene, glycoprotein B gene and the *EBV* (human herpes virus 4) non-glycosylated membrane protein (BNRF1) gen and *M. tuberculosis* MPB64/IS6110 repeated genomic sequence, were tested with the Genesig Advanced Kit (Primerdesign Ltd., York House, School Lane, Chandler’s Ford, United Kingdom) by following the instructions of the manufacturer. These tests have previously shown good sensitivity and specificity for diagnosis of herpes virus infections in humans [20]. Results of the qPCR were expressed as cycle thresholds or Ct values. All amplifications were performed using an Applied Biosystems Step One plus qPCR system.

All serum samples were analyzed for anti-*Toxoplasma* IgG and IgM antibody titers by using ELISA commercial assays and following the manufacturer’s (Human, Gesellschaft für Biochemica und Diagnostica mbH Max-Planck-Ring 21, 65,205 Wiesbaden, Germany) recommendation. Those with positive *Toxoplasma* IgG results underwent local ocular antibody production testing to calculate the GWC. This index was calculated by measuring the levels of intraocular anti-*Toxoplasma* antibodies, as described previously [16]: anti-*Toxoplasma* IgG in AH/total IgG in AH/anti-*Toxoplasma* IgG in serum/total IgG in serum [16].

Patients were also submitted to measurement of IgG and IgM in serum for HSV1, HSV2, VZV, CMV and EBV. Those with positive IgM results were considered to have active viral infection and were given specific treatment.

### Diagnostic flowchart, classification of diagnosis and clinical follow-up

**Fig. 1:**
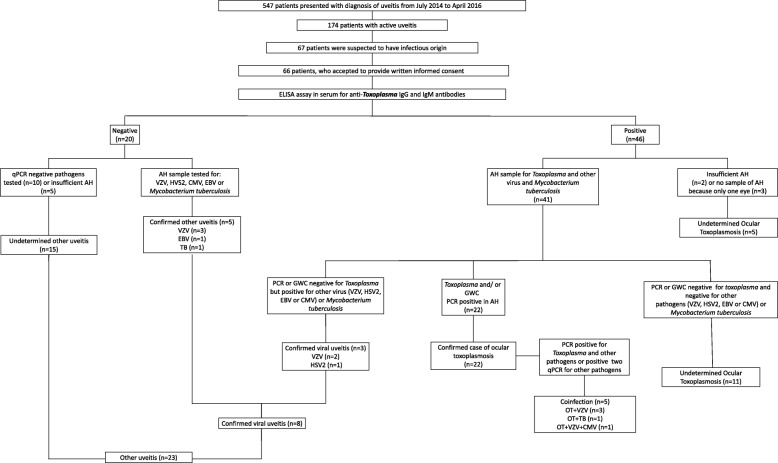
Diagnostic flowchart, classification of diagnosis and clinical follow-up. Flow chat for the diagnosis of confirmed ocular toxoplasmosis, undetermined toxoplasmosis, without ocular toxoplasmosis and co-infections: Specific PCR and GWC analyses in aqueous humor samples

We used a diagnostic algorithm that was previously used in French patients to unequivocally confirm OT in up to 80% of cases [16]. This consisted in the analysis of AH samples for diagnosis of *Toxoplasma* or other infections (Fig. 1). If local IgG production was detected (GWC < 2), or if PCR analysis in AH detected parasitic DNA, then the clinical diagnosis was considered to confirm OT. If no local specific IgG production or PCR was negative or an aqueous humor sample was not available, but the lesions were characteristic of toxoplasmosis and anti-*Toxoplasma* IgG antibodies were positive in serum, the diagnosis was considered as undetermined ocular toxoplasmosis (UOT). If anti-*Toxoplasma* IgG antibodies were negative in serum or an AH sample was not available and PCR was positive in AH for other etiologies, the diagnosis was considered as other uveitis (OU). If two or more PCR were positive for different pathogens in the same sample then it was considered a case of co-infection (COINF). The primary outcome measure was the frequency of OT, UOT, OU and COINF. Secondary outcomes were change in treatment based on the laboratory results and if improvement was observed after change of treatment or without change of treatment, during follow-up. Improvement after therapy was defined as recovering one or more lanes in visual acuity (using any measure) at the end of follow-up and reduction in retinochoroidal lesion size after 6 weeks of treatment. Patients were followed up closely to determine treatment response in terms of reduction of anterior chamber cellularity, vitreous cells and haze, reduction in lesion size, if applicable, and improvement in visual acuity.

### Statistical analysis

A data collection instrument was created for this study and entered into the database in Excel (Additional file 1) for subsequent statistical analysis using SPSS v14.0 (IBM, Armonk, New York,USA). Results were expressed as the median [min-max] for continuous variables and N (%) for categorical variables. Differences in proportions were analyzed using the chi-square test or Fisher’s exact test, when appropriate. Differences in means were compared by ANOVA or a non-parametric test if not normally distributed. Values below *p* < 0.05 were considered statistically significant.

## Results

Five hundred forty-seven patients presented with the diagnosis of uveitis from July 2014 to April 2016, 174 with active uveitis, from which 67 patients were suspected to have uveitis of infectious origin; and 66 patients, who accepted to provide written informed consent, were included in the study period.

Sixty-six consecutive patients – 32 females (47.8%), and 34 males (51.5%), aged between 13 and 79, with a median age of 37 years – who presented with uveitis that was clinically consistent with OT were first studied by *Toxoplasma* serological tests. According to the results of serum tests, specific *Toxoplasma* PCR and GWC analyses were performed in AH samples obtained by diagnostic anterior chamber paracentesis (Fig. 1). There were 22 cases of confirmed OT, eight of confirmed OU and five COINF. In total, 35 patients (53%) had etiological diagnosis confirmed by PCR or GWC (Fig. 1). OT was conclusively diagnosed for 27 patients through examination of aqueous humor samples (five of them with COINF): 9 tested positive on both tests (PCR and GWC) in aqueous humor; in 15 patients diagnosis was made by the high load of specific antibodies in aqueous humor alone and in three by the PCR alone. In confirmed OT, 12 out of 27 patients with PCR analysis were tested positive by PCR (44.4%) and 24 out of 27 with GWC analysis (88.8%) were found to be positive.

No significant differences in demographic and clinical characteristics were found between the group of patients according to their diagnoses, except for frequency of anterior uveitis that was only observed in OU or in unconfirmed toxoplasmosis (Table 1). In 21 patients (31.8%), there was a change of treatment after diagnosis. As expected, the change of treatment after diagnosis was significantly greater for the group of patients with OU and with COINF (Table 1). For the group of patients with OU, when treatment was changed from topical anti-inflammatory therapy to systemic anti-viral therapy, anti-TB therapy or to immunomodulatory therapy, according to the final diagnosis, improvement was observed in 12 out of 13 patients (92,3%). All of the patients with diagnosis of viral uveitis improved after anti-viral treatment. In contrast, improvement was observed in six out of eight patients without change in therapy after diagnosis (75%): however, this difference was not statistically significant (Fisher test *p* = 0,53). Coinfected patients were closely followed up, treated with the specific therapy for each case. Acyclovir was added in patients with VZV (*n* = 3) and anti-TB therapy was added in the case of Toxo+TB coinfection (*n* = 1) with improvement and resolution of the uveitis. In the case of triple coinfection, Toxo+VZV + CMV (n = 1) valacyclovir was prescribed in addition to the anti-*Toxoplasma* treatment, with improvement but not resolution of the inflammation, due to the unavailability of valganciclovir therapy. This patient persists with low grade of inflammation.

**Table 1:** Clinical characteristics of patients with uveitis according to final diagnosis

Characteristics of patients	Median (range) or number/total (percent) in COT patients (*n* = 22)	Median (range) or number/total (percent) in UOT patients (*n* = 16)	Median (range) or number/total in OU patients (*n* = 23)	Median (range) or number/total in COINF patients (*n* = 5)	p
Age	37,5 (13–70)	30 (19–74)	34 (17–79)	50,2 (16–79)	0,523
Gender Female	10/22 (45,5%)	6/16 (37,5%)	14/23 (60,9%)	2/5 (40%	0,492
Localization of uveitis (anterior, posterior and panuveitis)	0/22 (0%) anterior 12/22 (54,5%) posterior 10/22 (45,5%) panuveitis	1/16 (6,3%) anterior 14/16 (87,5%)% posterior 1/16 (6,3%) panuveitis	5/23 (21,7%) anterior 9/23 (39,1%) posterior 9/23 (39,1%) panuveitis	0/5 (0%) anterior 1/5 (20%) posterior 4/5 (80%) panuveitis	*0,004*
Bilateral involvement	3/22 (13,6%)	2/16 (12,5%)	7/23 (30,4%)	2/5 (40%)	0,351
Cataract	6/22 (27,2%)	4/16 (25%)	8/23 (34,7%)	1/5 (20%)	0,655
Papillitis	3/22 (13,6%)	1/16 (6,3%)	4/23 (17,4%)	1/5 (20%)	0,539
Total number of scars (both eyes)	2,5 (1–11)	2 (1–18)	1,5 (1–4)	2,5 (2–3)	0,800
Number of inflammatory cells in vitreous humor	2 (0–4)	0,5 (0–4)	1 (0–4)	2 (0–4)	0,403
Median size of scars in disk diameters	1,53 (1–6)	1 (1–4)	0,9 (1–3)	1,3 (1–2)	0,157
Median number of recurrences episodes	1,81 (0–10)	1 (0–4)	1,52 (0–10)	0,6 (0–2)	0,658
Number (%) of IgM serological test positive for virus (EBV, CMV, HVS1, HVS2)	2/22 (9%)	2/16 (18,8%)	5/23 (21,7%)	0/5 (0%)	0,716
Number of patients with intraocular pressure > 20 mmHg	4/22 (18,2%)	1/16 (6,3%)	2/23 (8,7%)	1/5 (12,5%)	0,236
Number of patients (%) with change of treatment after diagnosis	3/22 (13,6%)	1/16 (6,3%)	13/23 (56.5%)	3/5 (60%)	*0,002*
Significant clinical improvement after treatment change	3/3 (100%)	1/1 (100%)	12/13 (92,3%)	3/3 (100%)	–
Significant clinical improvement after 6 months of treatment (regardless of whether there was change or continuation)	21/22 (95.4%)	14/16 (87.5%)	20/23 (86.95%)	4/5 (80%)	–
IgG for Toxoplasmosis: qualitative	22/22 (100%)	16/16 (100%)	3/23 (13%)	5/5 (100%)	–
IgM for Toxoplasmosis: qualitative	3/22 (13.6%)	0/16 (0%)	1/23 (4.34%)	0/5 (0%)	–
IgM for EBV: qualitative	2/22 (9%)	2/16 (33.3%)	3/23 (13%)	0/5 (0%)	–
IgM for CMV: qualitative	0/22 (0%)	0/16 (0%)	0/23 (0%)	0/5 (0%)	–
IgM for HSV-1: qualitative	0/22 (0%)	0/16 (0%)	2/23 (8.6%)	0/5 (0%)	–
IgM for HSV-2: qualitative	0/22 (0%)	0/16 (0%)	0/23 (0%)	0/5 (0%)	–

*COINF* Co-infection, *COT* Confirmed ocular toxoplasmosis, *OU* Other uveitis, *UOT* Undetermined ocular toxoplasmosis

*statistically significant difference (*p*< 0,05)

Patients with UOT (*n* = 11) because of insufficient AH sample (*n* = 2) or because they did not undergo AH sampling (*n* = 3), might have been diagnosed with viral uveítis and/or ocular toxoplasmosis if AH could have been tested.

Considering the clinical signs, panuveitis was more common in patients with COINF, than in patients with UOT (80% vs 6.3%). While posterior uveitis was more common in patients with UOT (87% vs 20%), the presence of anterior uveitis was more frequent in patients with OU including viral origin than in patients with UOT or patients with COINF (21.7%, vs 6.3% vs 0% respectively). The specific clinical signs are shown in Table 1.

Of interest, IgM serological tests for the herpesvirus family (HSV1, HSV2, HSV3 and EBV) were positive without relation to the presence of the virus as detected by qPCR in aqueous humor (Table 1). In total, 9 patients were positive for these IgM assays (7 for EBV and 2 HSV1). One patient was positive simultaneously for IgM anti- HSV1 and EBV. Patients with IgM for several viruses might have had recent infection with one of them, inducing cross-IgM detection. Nevertheless, in our study none of them presented positive anti-Toxoplasma IgM. Also, none of these patients had positive serum tests for syphilis, thus we did not include aqueous humor PCR for *Treponema pallidum* in the patients. The five COINF patients were further investigated by IgM for CMV, and none of them had positive results.

OT was conclusively diagnosed in 27 patients through examination of AH samples (five of them in COINF): 18 tested positive on both tests (PCR and GWC) in AH; six diagnoses were made by the high load of specific antibodies in AH alone and three by the PCR alone. In confirmed OT, 12 out of 25 patients with PCR analysis were tested positive by PCR (48%) and 24 out of 26 with GWC analysis (92.3%) were found to be positive. Concerning the positivity of GWC and PCR according to the days of symptoms at the time of sampling and degree of inflammation, there were no statistically significant differences between positivity of AH PCR or of the GWC (Tables 2 and 3). A *Toxoplasma* IgG avidity test was performed in 44 patients with positive IgG titers. All the results of avidity were higher than 30%, indicating chronic infection acquired more than 4 months ago, including the four patients with a positive anti-*Toxoplasma* IgM test.

**Table 2:** Results of GWC/PCR in patients with OT or UOT, according to the time elapsed since the onset of symptoms and to cell counts in AH

Characteristics of patients with OT	GWC positive	GWC negative	*p* value (Kruskall Wallis test)	PCR positive	PCR negative	*p* value (Kruskall Wallis test)
Duration of symptoms before sampling in days. Median [range]. Number of patients with data and results of test (n)	30 [2–230] *n* = 21	25 [2–277] *n* = 8	0.8261	48 [1–277] *n* = 11	27 [2–230] *n* = 18	0.6692
Number of cells/μL of AH. Median [range] Number of patients with data and results of test (n)	2 [0–4] *n* = 24	1 [0–4] *n* = 15	0.5066	1.25 [0–3] *n* = 12	2 [0–3] *n* = 28	0.1903

*AC* Anterior chamber, *GWC* Goldmann–Witmer coefficient, *AH* Aqueous humor, *PCR* Polymerase chain reaction

**Table 3:** GWC/PCR in AH in confirmed OT according to the onset of symptoms and the treatment before sampling

Characteristics of patients with OT	GWC+/PCR+	GWC+/PCR-	GWC-/PCR+
Duration of symptoms before sampling in days. Mean ± SD. Number of patients with data and results of test (n)	48.6 ± 51 *n* = 8	55.2 ± 67 *n* = 12	163 ± 160 *n* = 2
Duration treatment before sampling (days). Mean ± SD. Number of patients with data and results of test (n)	17.3 ± 11.3 *n* = 6	21.2 ± 18.3 *n* = 5	- *n* = 0

*GWC* Goldmann–Witmer coefficient, *PCR* Polymerase chain reaction

2 patients GWC + and no PCR data. 1 patient PCR + and no GCW data. (Not included in the table)

Intraocular pressure was very high in eight patients (12%). One of these patients with COINF was initially diagnosed as typical recurrent OT, with a bad response to conventional treatment; this patient presented OT + VZV COINF. Another patient with triple COINF (TO+VZV + CMV), had bilateral compromise and did not have any history or clinical or laboratory evidence of immunodeficiency.

## Discussion

Diagnostic doubts are a factor in the management of uveitis [15, 21–24]. There are several differential diagnoses that must be considered when dealing with patients with suspected infectious uveitis [15, 21–24]. Several studies have demonstrated that by combining PCR and GWC the diagnostic certainty for toxoplasmic retinochoroiditis is increased, reaching 80–93% sensitivity and specificity of ~ 93% [23]. In our study, we made an accurate diagnosis of uveitis causes using GWC and PCR in 53% of our patients. The frequency of PCR positivity was similar to that reported in previous studies [16, 25, 26]. To the best of our knowledge, there are no previous studies about the presence of COINF in immunocompetent Colombian patients with OT. In the present work, combined laboratory techniques, in serum and AH, were of significant diagnostic value for patients diagnosed as having other intraocular infections (VZV, EBV, tuberculosis).

GWC is based on the comparison of levels of specific antibodies in ocular fluid and serum samples [26]. It has been reported in a study in French patients that the time interval from symptoms onset to anterior chamber tap is highly correlated with the GWC positivity. In our cases, there were no differences in days of symptoms in patients with positive or negative PCR; a similar result has previously been reported [26]. This could be explained because *Toxoplasma* infection is caused by different strains in Colombia and Europe. The load of parasites is higher in Colombian patients compared with European patients and this can induce a rapid increase in antibodies [27]. Colombian strains are usually known as more virulent, triggering a higher intraocular inflammatory response [28]. This might influence the delay or intensity of DNA detection or antibody detection in AH. The different strains explain why test results and clinical decisions vary according to the geographical origin of the ocular infection [29]. Our group in Colombia has consistently demonstrated that South American strains producing ocular toxoplasmosis are different and induce a different humoral response with different cytokine mediators [30]. This can help to understand why diagnostic performances of serological and molecular tests differ [31]. It also has been proven that the performance of RE target for PCR amplification is different when used in South American patients [29, 32, 33].

Patients with UOT (*n* = 16) were treated with anti-*Toxoplasma* therapy. All of them had a good clinical response. This is consistent with the traditional practice around the world (treating patients with suspected clinical picture of OT with positive serological anti-*Toxoplasma* tests). If the outcome was favorable under anti-*Toxoplasma* therapy, then patients could be definitely considered as OT even with aqueous humor negative. PCR is used only in patients with atypical clinical presentations or in patients with bad response to anti-*Toxoplasma* treatment [34, 35]. However, in our study we carried out these diagnostic techniques in patients with typical and atypical presentation. We have to take into account that a negative PCR result for *Toxoplasma gondii* does not rule out the diagnosis and that, in these cases, the clinical response to therapy is crucial to confirm the diagnoses [35]. Although there were no typical granulomas in the posterior pole suggesting ocular infection by *Toxocara canis* in our patients, toxocariasis was ruled out serologically when patients presented posterior uveitis or panuveitis and none of the patients screened had positive antibodies. Although Lyme disease is also an infectious cause of uveitis, it was not screened for, because there are not cases of Lyme disease reported as a cause of uveitis in Colombia and patients did not have any traveling history to the northern hemisphere. Thus, differential diagnosis by PCR in AH was not done for toxocariasis or Lyme disease [36] . As it cannot be excluded that OT was not diagnosed due to lack of sensitivity of the PCR or GWC, we merge the 3 patients with positive serological assay for *Toxoplasma* with the 20 patients with “other uveitis”. Considering that *Mycobacterium tuberculosis* and *Leptospira* [37] can also cause uveitis, we prefer to keep the term as “Other Uveitis (OU)” instead to consider cases of other uveitis only as of viral origin. This population was clinically homogenous regarding the characteristics of uveitis, leading as to think in infectious origin. It is necessary to improve the sensitivity of diagnostic techniques in AH in order to get definitive and conclusive diagnosis in these patients.

Multiple infectious diseases can be associated in immune-deficient patients with human immunodeficiency virus infection and acquired immune deficiency syndrome (HIV/AIDS), presented as simultaneous or separate infections leading to severe intraocular compromise [38, 39]. In our study, all patients were immunocompetent, including those with co-infection. It has been reported that EBV-DNA was found in HIV negative immunocompromised patients with uveitis. However, authors hypothesized that EBV is not a direct cause of uveitis, but it may play a role as a secondary factor in the pathogenesis of uveitis, producing a homologue of IL-10. This interleukin is known as an immunosuppressant that influences the course of intra-ocular inflammation caused by other pathogens as *Toxoplasma*, HSV, VZV and CMV [40].

CMV has been associated with anterior, chronic, hypertensive, unilateral and recurrent uveitis in immunocompetent patients [41]. In our work, we did not find CMV infection as a single cause of uveitis in this group of patients. We found CMV infection in coexistence with OT + VZV in one immunocompetent patient. To the best of our knowledge, there are no previous reports about triple intraocular co-infection in the literature in immunocompetent patients. Intraocular COINF of OT associated with viral infection was biologically proven in our patients. COINF presented in immune-competent patients deserves careful consideration. For instance, in a recent study, three immunocompetent patients were AH PCR double positive, for VZV + EBV, *Toxoplasma* + EBV, and T*oxoplasma* + HSV [18]. In these double positive cases, therapy was decided according to the clinical criteria, with close follow-up. Double positive PCR has previously been reported in immunosuppressed patients: it is probably due to secondary reactivation of latent parasitic or viral infections triggered by acute infectious uveitis. In the positive cases for EBV, we have to consider that the EBV genome could be present in a latent phase in B lymphocytes, leading to cross reactivity, which can produce false PCR positive results [42]. In immunosuppressed patients, PCR testing for multiple pathogens in ocular fluids can be useful for selecting treatment, since clinical characteristics could be atypical in these cases [43].

PCR in AH samples, GWC and a differential diagnosis assisted with therapeutic trials have been studied by other authors in anterior uveitis [44]. CMV was identified by PCR in aqueous and vitreous samples as the most frequently recognized infectious organism in posterior and panuveitis of HIV-1-negative Thai patients [45]. In our study, none of our COINF patients presented any associated systemic diseases or alterations in ancillary tests. Thus, we excluded primary or secondary immunodeficiencies.

It has been reported that intraocular immune response in OT differs depending on the infecting *Toxoplasma gondii* strain [46]. Virulent parasites, such as South American strains, may cause more severe OT due to an inhibition of the protective effect of interferon gamma (IFN-γ) [46]. IFN-γ is crucial in the immune response to viral diseases, providing antiviral protection from CMV infection [47]. There is also an important antiviral role for virus-specific CD4(+) T cells in protecting from pathogenic CMV infection [47]. In Colombian patients with OT, the immune response after antigenic stimulation has been found to have a preferential Th2-skewed response, regardless of the nature of antigen stimulus [48] and more severe clinical characteristics in patients infected by type I/III strains [30]; strain-dependent Th2 skewed response should be investigated to identify if it plays a role in favoring this type of co-infections. The Th2-skewed response in patients infected by virulent *Toxoplasma gondii* strains might have a detrimental role in the defense against viruses, aiding parasites and viral co-infection. T-helper response induction may be confounded by co-infection of a single host by multiple intracellular pathogens. Due to normally adaptive feedback loops that tend to polarize T-helper responses, it can become very difficult for the immune system to mount effective, conflicting responses [48].

## Conclusions

Ocular fluids PCR is useful in the diagnosis of uveitis with unusual clinical appearance, severe recurrent intraocular inflammation of unclear origin, and therapy-resistant patients. Additional studies are necessary to analyze how the co-infection affect the therapeutic response and prognosis in this group of patients, and to investigate the need to include additional tests. In future studies, additional molecular analysis for multiple pathogens in aqueous humor would be needed in order to determine if some etiologies were dismissed.

### Possible bias

The use of GWC instead of WB might reduce the sensitivity of the diagnosis of infection uveitis. GWC was used because we do not have Western-blotting currently commercially available in Colombia, it should be of interest to test this technique in the future to examine if this can increase the diagnostic performance in these cases.

Five patients lack data from AH tests; 2 because there was insufficient AH volume sample and 3 with only one functional eye who did not undergo AH sampling.

## Additional file

Additional file 1: Availability of Dataset. (XLSX 50 kb)

## Abbreviations

AH: Aqueous humor
BCVA: Best corrected visual acuity
CMV: Cytomegalovirus
COINF: Case of co-infection
EBV: Epstein-Barr virus
GWC: Goldmann–Witmer coefficient
HIV/AIDS: Human immunodeficiency virus infection and acquired immune deficiency syndrome
HSV1: Herpes simplex virus type 1
HSV2: Herpes simplex virus type 2
IFN-γ: Interferon gamma
IgG: Immunoglobulin G
IgM: Immunoglobulin M
OT: Ocular toxoplasmosis
OU: Other uveitis
PCR: Polymerase chain reaction
qPCR: Real-time polymerase chain reaction
SUN: Standardization of uveitis nomenclature
TB: Tuberculosis
UOT: Undetermined ocular toxoplasmosis
VZV: Varicella zoster virus
